# Miss Cobbe's Doctors

**Published:** 1889-11-30

**Authors:** 


### The Hospital

A Weekly Journal of

**Science, Medicine, Nursing, and Philanthropy.**

*For Hospitals, Asylums, Medical Practitioners, Students, Nurses, Families, Parishes, Congregations, and the Charitable Public.*

Vol. VII. No. 166.] [November 30, 1889.

### Miss Cobbe's Doctors.

Miss Frances Power Cobbe, as all the world knows, has a "pretty wit" and a caustic pen. Perhaps she does not like doctors in any shape! We know she does not like them when they take the form of experimental physiologists. Miss Cobbe is a clever woman, and, what is more, a good woman; and her very decided dislike of certain classes of doctors cannot be set down as all to their credit. The immediate instinct of a Briton who is struck is to strike back, and not to reflect whether he has deserved the blow or not. We shall endeavour to be so much superior to the average Briton in this particular as to take Miss Cobbe's last blow meekly, and to consider why she has struck it, and whether it is deserved.

Of late the philosophers of town councils and the amateur sanitarians of various talking congresses have been much exercised in mind about the new Act for securing the notification of infectious diseases. That is all very well, says Miss Cobbe, so far as it goes; but what about the notification of infectious doctors? A most pertinent question, and one to which doctors at any rate should feel anxious to give a prompt and, if possible, satisfactory reply. "How does it happen," says this keen-witted lady detective, "that the zeal recently directed to the protection of the public from infectious diseases should have stopped short at isolating the patients, who at worst are passive in their sick beds, and have taken no account of the doctors, who run all over the town, carrying, according to their own theories, millions of disease germs on their persons and clothing? It appears to be not a little inconsistent to pass an Act of Parliament invading the liberty of the subject and the sacred rights of parents and wives, all for the sake of securing safety from infection from a peaceful patient in his bed, and at the same time allow the doctors, who have been hanging over and touching him, to carry everywhere and spread broadcast, without let or hindrance, the germs of his disease. Such lop-sided legislation is on a par with the recent Bill before Parliament, which provided muzzles for lapdogs but allowed packs of foxhounds to go free." By way of emphasising and bringing home her contention to the most apathetic mind, Miss Cobbe relates a very grievous experience of her own. "Some years ago," she says, "a medical man of high standing, being summoned to attend a near relation of mine, thought fit to do so straight from a case of scarlet fever. The poor lady developed the disease immediately after the birth of her child, who, of necessity, was taken from her and conveyed to the best nurse I could find on the spur of the moment. The husband caught the fever from his wife, and her sister from one or other, and for a long time the lives of all three and of the infant hung on a thread. For all the misery, suffering, anxiety, and expense entailed by the shameful carelessness of the doctor, the law afforded no redress whatever."

That is Miss Cobbe's case. What have medical apologists to say in reply? Undoubtedly many of them must first plead guilty; all must express their sorrow; and all, without exception, must promise to walk in more scientific as well as in more just and brotherly ways for the future. The non-medical public will probably feel not a little surprised that any doctor of high standing should go directly from a case of scarlet fever to attend a case of confinement. The truth, however, is simply this, that familiarity breeds contempt in doctors exactly as it does in other people. The particular medical man mentioned by Miss Cobbe had probably attended hundreds, perhaps thousands, of cases of scarlet fever, and immediately thereafter had made other calls, without having been able in a single instance to trace any bad results to the practice; and therefore, in spite of his knowledge of the powerfully infectious character of scarlet fever, he had come to feel in his own mind that there was no really serious risk in what he was doing. Probably, too, he was sent for in a great hurry; and it may be that the patient in that case, as patients so often are, was very averse to being attended by any other medical man. All this of course is of the nature of special pleading, and by way of making the best conceivable apology for what was in itself a wrong and wicked thing, and a thing that it ought not to have been possible for any intelligent doctor to have permitted himself to do.

But the real interest of Miss Cobbe's attack lies in that part of it which demands protective legislation for the patients of medical men; and the infliction of adequate penalties in cases of proved fault. There are practitioners, undoubtedly, who will consider the mere suggestion of legislation of this kind a grievous hardship and injury. Seeing clearly their own side of the question, and knowing how easy it is for ill-disposed persons to make charges of culpable carelessness against a medical man, and how difficult it is to prove the baselessness of such charges when made, they stand aghast at the idea of Parliament putting into the hands of all and sundry a weapon of such terrible effectiveness as Miss Cobbe's proposed Act of Parliament might prove itself to be. Therein our *confrères* have our most profound sympathy. But it must be remembered that there is another aspect of the question as well as the medical. The public has its case to put forward; and if a medical man really desires to take the right view of the matter, he will, in imagination, divest himself forthwith and completely of all the attributes that are peculiar to him as a doctor, and for the nonce became a member of the lay public. Looking at himself as a patient rather than a doctor, and at his family as the patients of the most careless medical man he knows, he will find that there is a great deal —a very great deal—to be said for Miss Cobbe's plea.

It is probable that legislation on the lines of Miss Cobbe's contention will come, and that in no long time. But if we are to expect that the public will insist upon being legally protected against what she considers the "shameful carelessness" of doctors, it is worth while considering what steps it may be proper for doctors to take to protect themselves against what may often prove to be the "shameful vindictiveness" of some of their clients. For our part, as we have said, we see no good reason why an Act should not be passed for the punishment of any medical man, whatever his standing, who neglects to take the most thorough and scientific precautions against the possibility of his acting as a medium of contagion for infectious diseases. But whilst we are most anxious that the public should be protected, we are equally desirous that medical men themselves should run no risk of being ruined in character and finances by the rash and vengeful feelings of disappointed and dissatisfied clients.

What, then, must doctors do to protect themselves? Is it desirable in the least degree to seek special legal protection? By no means. Lord Salisbury has taken frequent opportunities of expressing his contempt for those who believe that human ills can be largely cured, or human progress largely advanced by legislation. We entirely agree with him. There can be no more unworthy line of conduct for any class of men to pursue than that of invoking the Legislature to do for them what they can and ought to do for themselves. And such conduct is as futile as it is unworthy. There is a method by which medical men may protect themselves against the incidence of any legislation which may be made in the interest of the public safety, a method so simple that we almost hesitate to announce it. It is the method of always doing to patients exactly as they would like to be done by if they were patients. A medical man has no right to subject any patient to the least shadow of risk from him. Nothing can be more obvious than this, that a doctor, of all persons, should be in such a condition of wholesomeness as to his person and his clothes, that the most delicate infant might play with him with perfect safety. For a doctor to be a source of infection, and to defend himself in it, is the same as if a fireman should carry dangerous combustibles into houses that are not burning, and should plead that as a fireman he is justified in so doing. All modern science points to the conclusion that infectious germs are numerous and readily transmissible. It also points to the fact that such germs, as a rule, are easily destroyed and made harmless. The public has the clearest possible right to demand at the doctor's hands that he shall use the methods of making those germs harmless which are so well known and so conveniently available. Doctors who think and act otherwise, have yet to learn the very elements of that ethical code which should constantly rule their conduct in this as in every relation of life.

